# Functionalized 3D-Printed PLA Biomimetic Scaffold for Repairing Critical-Size Bone Defects

**DOI:** 10.3390/bioengineering10091019

**Published:** 2023-08-29

**Authors:** Xiao Liu, Jianpeng Gao, Xiang Cui, Shaobo Nie, Xiaoyong Wu, Licheng Zhang, Peifu Tang, Jianheng Liu, Ming Li

**Affiliations:** 1 Medical School of Chinese PLA, Beijing 100853, China; liuxiao23_314@163.com (X.L.); bonegaojp@163.com (J.G.); 2 Department of Orthopaedics, The Fourth Medical Center of the Chinese PLA General Hospital, Beijing 100853, China; cuixiang0828@163.com (X.C.); nieshaobo1@126.com (S.N.); wxy1999_2008@hotmail.com (X.W.); zhanglcheng218@126.com (L.Z.); pftang301@163.com (P.T.); 3 National Clinical Research Center for Orthopedics, Sports Medicine & Rehabilitation, Beijing 100853, China

**Keywords:** three-dimensional printing, air plasma, type I collagen, simulated body fluid, bone defect repair

## Abstract

The treatment of critical-size bone defects remains a complicated clinical challenge. Recently, bone tissue engineering has emerged as a potential therapeutic approach for defect repair. This study examined the biocompatibility and repair efficacy of hydroxyapatite-mineralized bionic polylactic acid (PLA) scaffolds, which were prepared through a combination of 3D printing technology, plasma modification, collagen coating, and hydroxyapatite mineralization coating techniques. Physicochemical analysis, mechanical testing, and in vitro and animal experiments were conducted to elucidate the impact of structural design and microenvironment on osteogenesis. Results indicated that the PLA scaffold exhibited a porosity of 84.1% and a pore size of 350 μm, and its macrostructure was maintained following functionalization modification. The functionalized scaffold demonstrated favorable hydrophilicity and biocompatibility and promoted cell adhesion, proliferation, and the expression of osteogenic genes such as ALP, OPN, Col-1, OCN, and RUNX2. Moreover, the scaffold was able to effectively repair critical-size bone defects in the rabbit radius, suggesting a novel strategy for the treatment of critical-size bone defects.

## 1. Introduction

The incidence of critical-size bone defects due to trauma, infection, tumor, and severe osteoporotic fractures is increasing in parallel to the advancement of modern society [1,2]. According to statistics, approximately 500,000 patients undergo bone defect repair surgery each year in the United States and Europe, costing over $3 billion [3]. Furthermore, the expenses associated with secondary surgical interventions and subsequent hospitalization resulting from unsuccessful bone repair and reconstruction can reach up to $17,000–$79,000 per case, with a treatment duration that is three to five times longer and hospitalization costs that are four to twenty-five times greater [4]. Thus, the reconstruction of critical-size bone defects poses a significant challenge for contemporary orthopedic surgeons.

Recent advancements in bone tissue engineering techniques have enabled the emergence of novel treatment strategies for the mending of critical-size bone defects [5,6]. Three-dimensional printing technology is one such approach, which is used to fabricate biodegradable biomaterials based on predetermined bionic structures, enabling the rapid construction of bionic scaffolds possessing precise porous structures that accurately replicate the internal morphology and microstructure of human bone [7,8]. Fused deposition modeling technology is the most commonly used and mature technique, which involves printing high molecular-weight polymers according to a certain configuration and pore structure [9]. Studies have demonstrated that this technology can effectively facilitate cellular adhesion, proliferation, migration, and differentiation [10,11]. Moreover, porosity levels ranging from 55% to 80% have been found to be particularly effective in promoting the growth of new bone and providing adequate mechanical strength [12,13].

Polylactic acid (PLA) is a medically relevant polymer material synthesized from lactic acid, which is broken down into carbon dioxide and water via the tricarboxylic acid cycle [14]. It has a stable chemical structure, favorable plasticity and mechanical properties, and excellent biocompatibility [15]. Additionally, PLA is capable of controlled degradation without the production of toxic degradation products. The United States Food and Drug Administration approved its usage in the biomedical field as early as the 1970s, and it is still widely used in this domain [16]. However, the hydrophilicity deficiency of PLA material impedes the adhesion and penetration of cells onto the scaffold surface [17]. Additionally, the lack of biometric sites on the PLA surface hinders the generation of favorable interfacial reactions, leading to suboptimal osteogenic properties [18]. Therefore, attaining optimal critical-size bone defect restoration via 3D-printed PLA scaffolds alone is difficult, necessitating the incorporation of effective modification techniques.

The utilization of the plasma technique has the capacity to modify the surface properties and microstructure of PLA materials, such as roughness, morphology, charge, chemical composition, and surface energy, without altering the intrinsic properties of the PLA materials, thereby effectively augmenting their hydrophilicity and enhancing their biocompatibility [19]. Moreover, this technique facilitates the formation of cell recognition sites, promotes cell adhesion and proliferation, and increases biological recognition sites through the introduction of reactive groups, which in turn encourage protein adhesion and bone ingrowth [20,21].

Type I collagen, an essential extracellular matrix for bone composition, is known for its excellent biocompatibility and non-immunogenic properties, making it an effective agent for the repair of critical-size bone defects [22]. Similarly, hydroxyapatite minerals, a key component of bone inorganic material, have been found to be effective in promoting cell adhesion and proliferation [23]. In this study, a bionic surface structure comprising type I collagen and hydroxyapatite was created through a process of coating, grafting, and protein fixation onto the surface of 3D printed scaffolds following plasma modification. The efficacy of a bionic composite material in promoting critical-size bone defect repair was investigated by incorporating collagen to augment biorecognition sites and mineralization modification to construct a bone-apatite-like coating on the sample’s surface. This resulted in the development of a bionic surface structure that holds the potential to serve as a novel functionalized modification approach for the treatment and repair of critical-size bone defects.

## 2. Materials and Methods

### 2.1. Preparation of 3D-Printed PLA Porous Scaffold

The SolidWorks software 2021 SP2.0 (Dassault Systems SolidWorks Corp., Waltham, MA, USA) was used to fabricate the PLA porous scaffold via 3D printing. A cylindrical scaffold measuring 12 mm in length and 5 mm in diameter was created to investigate radial defect repair in rabbits, with approximate square holes of 350 μm × 350 μm in the anterior, superior, and right planes and an inter-hole spacing of 350 μm. A flat cylindrical scaffold with a diameter of 12 mm and a length of 3 mm was constructed for in vitro cellular experiments.

The STL format file containing the data model was imported into the printing software to generate the necessary code, which was then transferred to the CR-3040 3D printer (Creative 3D Technology Co., Ltd., CR-3040, Shenzhen, China). The 3D printer utilizing FDM printing technology was used to produce a 3D printed PLA scaffold with a uniform pore structure. This was achieved by printing the molten medical-grade PLA (Lvbao Biotechnology Co., Ltd., Shenzhen, China) in a layered and orderly manner at 210 °C, following the predetermined path with the basic printing parameters set as follows: a base layer filling thickness of 1 mm, a filling density of 20%, a printing speed of 30 mm/s, a nozzle temperature of 210 °C, a hotbed temperature of 50 °C, an extrusion volume of 100%, a nozzle aperture of 0.3 mm, and a bottom–up printing direction. The 3D-printed scaffold mentioned above has been designated as the P group.

### 2.2. Functionalization of PLA Porous Scaffolds

#### 2.2.1. Plasma Modification (PI Group)

The hollow plasma surface treatment system (YIHONG Technology Co., Ltd., CRF-VPO-8L, Wenzhou, China) was employed to modify the samples in an air atmosphere. During this process, the pressure was adjusted to 240 Pa, the discharge power was set to 20 W, and the control frequency was 13.56 MHz. The treatment time was 30 min, during which glow discharge plasma was generated to treat the samples.

#### 2.2.2. Collagen Infiltration (PC Group)

After plasma modification, with a 6-h interval, the samples were infiltrated with 2 mg/mL of type I collagen (Shi Feng Biotechnology Co., Ltd., Shanghai, China) for 24 h at 4 °C.

#### 2.2.3. Simulation of Body Fluid Mineralization (PS Group)

After plasma modification, with a 6 h interval, the PLA scaffolds were immersed in a simulated body fluid (SBF) for one day at 36.5 °C. The SBF was prepared in accordance with the methodology outlined by Kokubo et al. [24].

#### 2.2.4. Simulate Body Fluid Mineralization Following Collagen Infiltration (PCS Group)

After plasma modification with a 6 h interval, the PLA scaffolds underwent simulated body fluid mineralization following collagen infiltration, as described above.

After subjecting all material groups to various modification methods, the samples were rinsed with secondary water three times, followed by a 60 min disinfection process utilizing cobalt 60. Finally, the samples were sealed and stored at a temperature of 4 ℃.

### 2.3. Characterization Analysis

#### 2.3.1. Gel Permeation Chromatography (GPC)

The detection of molecular weight was conducted using a Waters 1515 gel permeation chromatograph (Waters Technology Co., Ltd., Shanghai, China), with full dissolution of the samples in chloroform (Green Protection Biotechnology Co., Ltd., Shenzhen, China). The parameters, including the number-average molecular weight (Mn, Dalton), the weight-average molecular weight (Mw, Dalton), the viscosity-average molecular weight (Mv, Dalton), and the molecular-weight distribution coefficient (d), were assessed.

#### 2.3.2. Nuclear Magnetic Resonance Spectroscopy (NMR)

The Av300 NMR spectrometer (Bruker, Germany) was employed to detect the thin-film H-atom species and number ratio. A solution of 5 mg of PLA powder in 0.5 mL of deuterium solvent was introduced into a standard NMR tube.

#### 2.3.3. Fourier Transform Infrared Spectroscopy (FTIR)

FTIR spectroscopy was performed on an Avatar-330 infrared spectrometer (Thermo Nicolet, Gilroy, CA, USA) with an accumulation of 32 scans from 400 to 4000 cm^−1^ and a resolution of 4 cm^−1^.

#### 2.3.4. Scanning Electron Microscopy (SEM) and Energy Dispersive Spectroscopy (EDS)

Upon affixing the PLA porous holder to the test sample table utilizing conductive double-sided tape, the sample table was subsequently introduced into the ion sputterer. The system was evacuated to a pressure of 10^−4^ Pa, coated with platinum, and subjected to scanning for sample analysis. The elemental composition of the designated region was subsequently verified.

#### 2.3.5. X-ray Photoelectron Spectroscopy (XPS)

An XPS analysis (AXIS NOVA, Tokyo, Japan) was conducted on the PLA powder compressed into a sheet with a pressure of 6 MPa for 1 min.

#### 2.3.6. Porosity Testing

Porosity testing was conducted to calculate the overall volume (Vw) of the scaffold, which encompassed both the scaffold and its internal air, by measuring the length (L), width (W), and height (H) of each scaffold as well as its mass (MS). Subsequently, the scaffold should be fully submerged in a density bottle containing anhydrous ethanol, which has a density of ρe and a mass of M1. The scaffold’s mass (M2) should then be reweighed. The porosity (ε) of the scaffold was determined using the following equation:

Vw = H × L × W

VS = (M1 − M2 + MS)/ρe

ε = 1 − Vs/Vw

### 2.4. In Vitro Cytology Testing

#### 2.4.1. Cell Culture

The MC3T3-E1 subclone 14 mouse embryonic osteogenic precursor cells were procured from the PLA General Hospital orthopedic laboratory for experimentation. The cells were expanded up to the 3rd generation using α-MEM medium supplemented with 10% fetal bovine serum and 1% penicillin–streptomycin. The cells were maintained in a controlled environment at 37 °C with 5% CO_2_, with the medium being replaced every other day. The cells were inoculated on the scaffolds at the indicated densities for subsequent experiments.

#### 2.4.2. Methyl Thiazolyl Tetrazolium Assay (MTT)

The MC3T3-E1 cells were inoculated with scaffolds at a density of 5 × 10^4^/well, and 600 μL of fresh medium and 24 μL of MTT solution (5 mg/mL) were added to each well on 24, 48, and 72 h. After a 4 h incubation period, 600 μL of DMSO was introduced to each well, followed by a 30 min incubation period at a constant temperature with uniform stirring for 10 min. The absorbance was then measured at 570 nm.

#### 2.4.3. Alkaline Phosphatase (ALP)

The MC3T3-E1 cells were inoculated in accordance with previously established protocols. On days 7, 14, and 21, 200 μL of cell lysis solution were added, followed by 200 μL of phosphate-buffered saline (PBS) solution after a 30 min lysis period. A total of 30 μL of the sample to be measured was then carefully added to the wells of a 96-well plate, while 30 μL of phenol standard application solution (0.02 mg/mL) was added to the standard wells and 30 μL of double-distilled water was added to the blank wells. The optical density (OD) values were measured at 520 nm after a 15 min incubation period with the addition of a chromogenic agent.

#### 2.4.4. Alizarin Red Staining and Quantification

The MC3T3-E1 cells were inoculated and subsequently treated with an osteogenic induction solution to sustain the culture. On days 14 and 21, the cells were fixed using a 4% paraformaldehyde solution for a duration of 20 min. Following this, the cells were washed three times with PBS and stained with alizarin red for 5 min for observation. Furthermore, 1 mL of 10% (*w*/*v*) cetyl pyridinium chloride dissolved in 10 mM sodium phosphate buffer, pH 7.0, was introduced into each well, and the optical density was measured at 490 nm after allowing the calcium salt to dissolve.

#### 2.4.5. Scanning Electron Microscopy (SEM)

Following a 24 h inoculation period of MC3T3-E1 cells, the cells were washed thrice with PBS, fixed with pre-cooled 2.5% glutaraldehyde at 4 °C for 20 min, and subsequently dehydrated with a gradient concentration of ethanol for 10 min. After another round of washing with PBS, the cells were subjected to CO_2_ critical point drying. The samples were then gold-sprayed and examined for cell morphology using SEM (EVO 18, ZEISS, Jena, Germany).

#### 2.4.6. Osteoblast Differentiation-Related Gene Testing

The MC3T3-E1 cells were inoculated following established protocols and incubated for 24 h in a standard medium. Subsequently, the medium was substituted with either osteoinductive medium containing 50 µg/mL ascorbic acid, 10 mM β-phosphoglycerol, and 10 nM dexamethasone or osteoinductive extracts. Following a 14-day culture period, the cells were subjected to total RNA extraction for real-time PCR analysis utilizing Trizol reagent (G3013, Servicebio, Wuhan, China). Each sample was replicated three times.

### 2.5. In Vivo Biological Testing

#### 2.5.1. Modeling of Critical Size Bone Defects

Thirty-six twenty-four-week-old female New Zealand rabbits (weighing (3.1 ± 0.9) kg) were randomly allocated into six groups of six rabbits each. Following successful anesthesia, a longitudinal incision of approximately 3.0 cm was made on the dorsal radius of the rabbit forelimb to create a radial defect measuring 1.2 cm in length. Following the implantation of sterile material, the wound was meticulously closed in a layered fashion. All rabbits were given intramuscular injections of penicillin at a dosage of 400,000 IU/day for a period of 7 days.

#### 2.5.2. X-ray Testing

The rabbits with critical-size bone defects underwent X-ray examination at intervals of 0, 4, 8, and 12 weeks postoperatively, using an X-ray fluoroscopic camera (Simens, Branford, CT, USA). The affected limb was exposed to X-ray irradiation at 120 kV, 80 mA, and 0.12 S following the administration of amiodarone intramuscular anesthesia.

#### 2.5.3. Micro-CT Testing

The Inveron MM System (Siemens, Munich, Germany) was employed to assess the quantity of newly formed bone in each rabbit group through micro-CT scans. The scanning parameters were set at 70 kV, 300 μA, 14.08 um pixel layer thickness, and 15 min of scanning time. Upon completion of the scan, the 3D spatial parameters, such as bone volume (BV), bone volume fraction (BV/TV), trabecular gap (Tb. Sp), trabecular thickness (Tb. Th), trabecular number (Tb. N), and bone mineral density (BMD), were analyzed using the Analyze 12.0 system.

#### 2.5.4. Postoperative Mechanical Testing

The mechanical compressor was used to apply a loading span of 15 mm and a fulcrum span of 30 mm to the radius, which was then compressed at a rate of 1 mm/min until fracture. The resulting load-deformation curve was documented. From this curve, the indices of maximum load (Max load), maximum strength (Max strength), elastic load (Elastic load), and elastic strength (Elastic strength) were determined.

#### 2.5.5. Histological Testing

Following a 48 h fixation period in 4% paraformaldehyde, the samples underwent decalcification with 10% EDTA, dehydration in graded concentrations of ethanol, and clearance with xylene. The samples were then embedded in paraffin and sectioned into 10 mm sections for HE, Masson, and Toluidine Blue staining via a microtome. Selected regions were examined under a microscope, and images were captured.

### 2.6. Statistical Analysis

Statistical analysis was conducted using GraphPad Prism 8 software (GraphPad Software, San Diego, CA, USA) and verified through the normality test. For parametric data, one-way ANOVA with Tukey’s post hoc test was utilized, while non-parametric data were analyzed using the Kruskal–Wallis test with Dunn post hoc. Significance levels were set at *p* < 0.05 (*), *p* < 0.01 (**), *p* < 0.001 (***), and *p* < 0.0001 (****).

## 3. Result

### 3.1. Characterization Analysis

**Figure 1:**
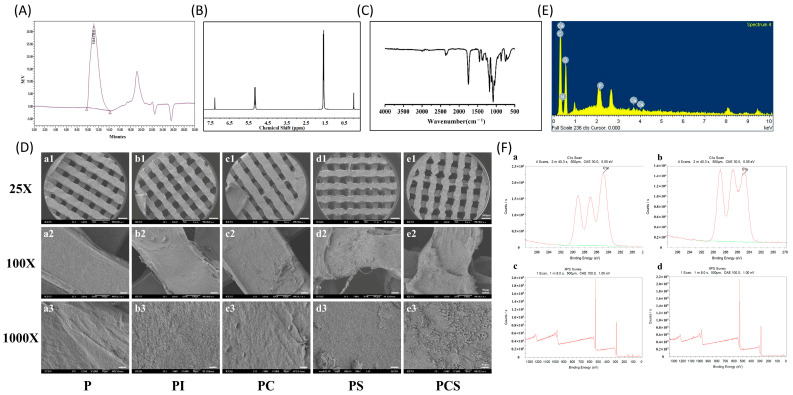
Characterization analysis: (**A**) Gel Permeation Chromatography (GPC) test results for PCS group scaffolds. (**B**) Nuclear Magnetic Resonance Spectroscopy (NMR) results of PCS group scaffolds. (**C**) Fourier Transform Infrared Spectroscopy (FTIR) results of PCS group scaffolds. (**D**) Scanning Electron Microscopy (SEM) results for different groups of scaffolds (the magnifications of 25×, 100×, and 1000× correspond to scale bars of 500 μm, 50 μm, and 10 μm, respectively). (**E**) Results of Energy Dispersive Spectroscopy (EDS) elemental content analysis of the PCS group scaffold. (**F**) C1s region energy spectrum and high-resolution X-ray Photoelectron Spectroscopy (XPS) energy spectrum (a and c: P group. b: PI group. d: PCS group).

The results of the GPC testing of the PCS group are presented in Figure 1A. The Mn, Mw, and Mv values were 80,019 (Dalton), 104,004 (Dalton), and 104,794 (Dalton), respectively. Figure 1B displays the NMR results of the PCS group, which indicate that the functionalized modification did not significantly impact the overall performance of the material. FTIR analysis results, shown in Figure 1C, suggest that FTIR curves reveal the presence of active functional groups such as C=O and -COO on the surface of PLA scaffolds after plasma modification. However, the modification did not significantly alter the overall properties of the PLA material. The SEM images revealed varying degrees of surface alteration on the PLA scaffold surfaces following modification. Under 25× microscopy, all scaffolds exhibited homogeneous porous structures with identical pore sizes (Figure 1(Da1–De1)). Upon closer observation (25× to 1000× microscopy), the P group displayed a smooth surface (Figure 1(Da1–Da3)), whereas the PI group had a significantly rough surface with a furrow-like pattern (Figure 1(Db1–Db3)). The group utilizing PC observed the presence of patchy structures on the surface of the material (Figure 1(Dc1–Dc3)), whereas the PS group exhibited a more pronounced surface roughness and the presence of punctate crystalline structures (Figure 1(Dd1–Dd3)). Comparable findings were observed in the PCS group, while its material surface displayed large raised-like structures on its rough surface (Figure 1(De1–De3)). Furthermore, elemental analysis was conducted on specific regions of the scaffold’s surface using SEM equipped with an EDS detection system. The findings revealed the presence of characteristic elements, including calcium (Ca), phosphorus (P), magnesium (Mg), and nitrogen (N), following functionalized modification of the material (Figure 1E).

The C1s scan of the P and PI groups exhibited three distinct peaks at binding energies of 284.6 eV, 286.4 eV, and 288.8 eV, which corresponded to the -C-H/-C-C-, -C-O-, and C=O/-COOH bonds (Figure 1(Fa,Fb)). Notably, the surface of the PI group demonstrated a significant increase in the -C-O- and C=O/-COOH bonds by 14.7% and 41.3%, respectively, compared to the P group. Conversely, the -C-H/-C-C- bonds decreased by 11.1%. The PSC group exhibited a greater presence of oxygen-containing reactive groups, and the immobilization of type I collagen led to a notable rise in nitrogen content. Following SBF incubation, a substantial quantity of calcium, phosphorus, and magnesium was detected in the surface elements of the material, thereby validating the effective modification of collagen loading with a hydroxyapatite-like coating (Figure 1(Fc,Fd)).

**Figure 2:**
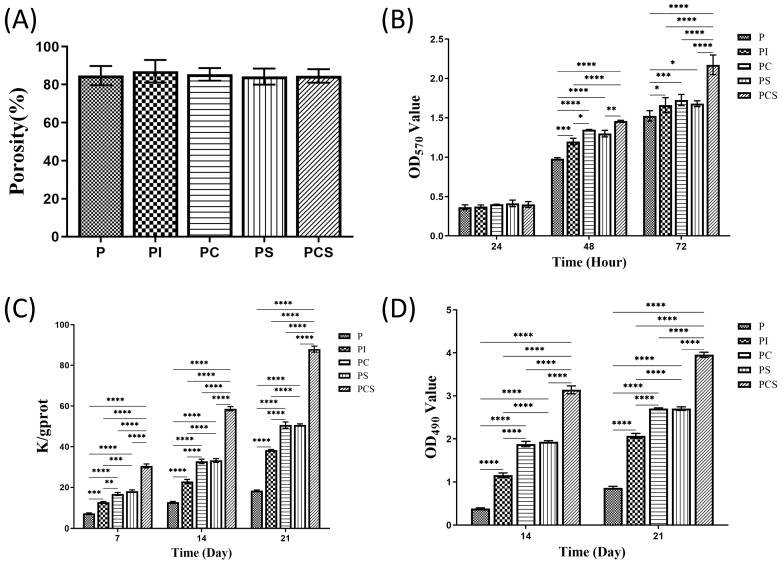
Characterization analysis: (**A**) Comparison of the porosity of five groups of supports measured by the volumetric method (n = 5). In vitro cytology testing: (**B**) MTT results of five groups of scaffolds with different functionalization modifications for the time periods of 24, 48, and 72 h. (**C**) ALP activity assay results for five groups of scaffolds co-cultured with MC3T3-E1 cells for 7, 14, and 21 days (the calculation for ALP viability (K/gprot) is derived from the formula: (assay OD—blank OD)/(standard OD—blank OD) × phenol standard concentration divided by the concentration of the protein sample being tested (gprot/mL)). (**D**) Quantitative analysis of Alizarin Red staining after co-culture of 5 groups of scaffolds with MC3T3-E1 cells at 14 and 21 days. The results were expressed as mean ± standard error of the mean (*n* = 3; significance levels were established as * *p <* 0.05, ** *p <* 0.01, *** *p <* 0.001, **** *p <* 0.0001).

All five groups of 3D-printed PLA scaffolds had a porosity greater than 80%. The scaffold porosity was measured to be (84.7 ± 5.1)% in the P group, (87.0 ± 6.0)% in the PI group, (85.4 ± 3.4)% in the PC group, (84.6 ± 3.7)% in the PS group, and (84.2 ± 4.3)% in the PCS group. No statistically significant differences were observed in the scaffold porosity among the five groups (Figure 2A).

### 3.2. In Vitro Cytology Testing

The MTT assay revealed the PCS group exhibited superior cell growth promotion capabilities when co-cultured with MC3T3-E1 cells compared to the other four groups of PLA scaffolds with distinct functionalized modifications. At the 24 h mark, no significant alterations or statistical variances were observed. At 48 and 72 h, the PCS group exhibited higher cell proliferation than the other groups, although the differences were not statistically significant (Figure 2B).

On day 7, the ALP activity was higher in all four groups of functionally modified PLA scaffolds compared to the P group, with the PCS group exhibiting the highest ALP activity. After 14 days of co-culture, it was evident that the PCS group continued to exhibit the highest ALP activity. The trend of ALP activity in the aforementioned groups remained unchanged when incubated for 21 days (Figure 2C).

**Figure 3:**
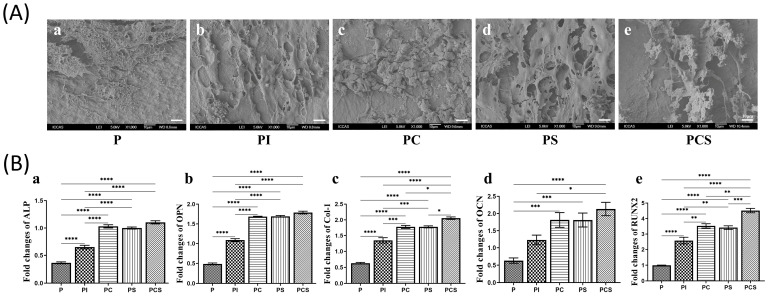
In vitro cytology testing: (**A**) Scanning Electron Microscopy (SEM) morphology observation after co-culture of five groups of scaffolds with MC3T3-E1 cells for 24 h (scale bar: 10 μm). (**B**) Osteogenesis-related gene expression in five groups of scaffolds after co-culture with MC3T3-E1 cells for 14 days (**a**) Alkaline phosphatase (ALP); (**b**) Osteopontin (OPN); (**c**) Collagen-1 (Col-1); (**d**): Osteocalcin (OCN); (**e**): Runt-related transcription factor 2 (RUNX2). The results were expressed as mean ± standard error of the mean (*n* = 4; significance levels were established as * *p <* 0.05, ** *p <* 0.01, *** *p <* 0.001, **** *p <* 0.0001).

Quantitative analysis of alizarin red staining conducted after 14 and 21 days revealed an increase in the calcium salt content with prolonged co-culture time, with the PCS group exhibiting the highest levels. The results from the PC and PS groups were comparable, suggesting that both modifications had similar effects on promoting cell differentiation. By contrast, the calcium salt formation in the PI group exhibited a slightly lower level than the other groups, with a statistically significant difference observed when compared to the P group. Additionally, the amount of calcium salt formation in the PI group was significantly higher than that observed in the P group, representing a statistically significant difference (Figure 2D). To detect the adhesion of MC3T3-E1 cells on various scaffold groups, SEM was utilized to observe cell morphology, pseudopod protrusion, and clustering on scaffold surfaces. The results indicated that cells cultured on the modified scaffolds exhibited a notable augmentation in pseudopod protrusion and adhesive clustering in comparison to the control group (P group) (Figure 3A).

The osteogenic differentiation potential was assessed through the expression of osteogenic-related genes, including ALP, OPN, Col-1, OCN, and RUNX2. Following a 14-day co-culture of cells with the aforementioned scaffolds, the PCS group demonstrated the highest expression levels of the ALP gene, while the PC and PS groups exhibited comparable expression levels. No significant differences were observed between the aforementioned three groups; however, their expression levels were significantly higher than those of the PI and P groups (Figure 3(Ba)). The expression of the OPN and Col-1 genes was comparable to that of the ALP gene, albeit with a higher overall expression content (Figure 3(Bb,Bc). The OCN gene exhibited the highest expression in the PCS group at 14 days, surpassing that of the PC and PS groups, although without statistical significance (Figure 3(Bd)). On the other hand, the RUNX2 gene demonstrated a significant increase in expression in the PCS group at 14 days, surpassing that of the PC and PS groups with statistical significance (Figure 3(Be)).

### 3.3. In Vivo Biological Testing

#### 3.3.1. X-ray Testing

**Figure 4:**
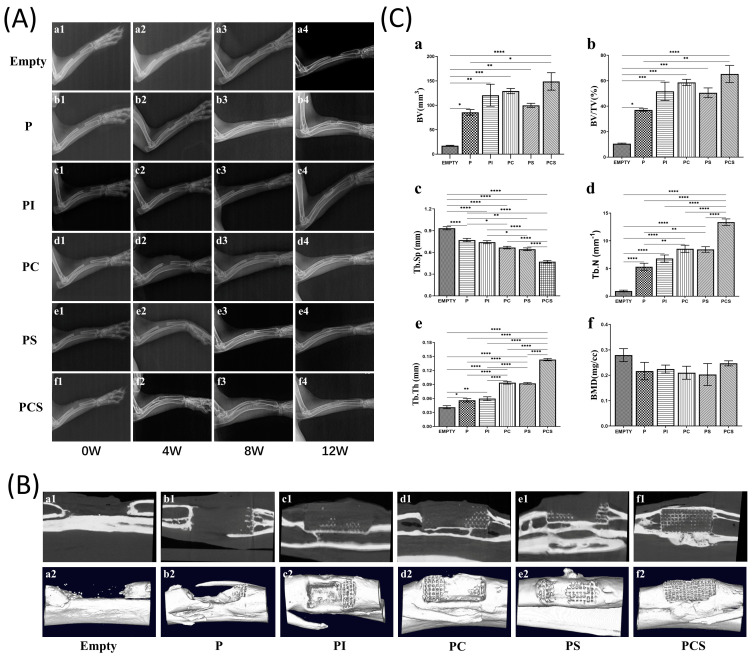
In vivo biological testing: (**A**) Radiographs of radial bone defects in rabbits from the blank control group and five groups with different functional modifications at different times (0, 4, 8, and 12 weeks) postoperatively. (**B**) Longitudinal micro-CT (**a1**–**f1**) and 3D reconstruction images (**a2**–**f2**) of radial defects in six groups of rabbits 12 weeks postoperatively. (**C**) Analysis of micro-CT radial critical-size bone defect parameters in six groups of rabbits at 12 weeks postoperatively: (**a**) Bone volume (BV); (**b**) Bone volume/Tissue volume (BV/TV); (**c**) Bone trabecular separation (Tb.Sp); (**d**) Bone trabecular number (Tb.N); (**e**) Bone trabecular thickness (Tb.Th); (**f**) Bone mineral density (BMD)). The results were expressed as mean ± standard error of the mean (*n* = 3; significance levels were established as * *p <* 0.05, ** *p <* 0.01, *** *p <* 0.001, **** *p <* 0.0001).

At 8 W postoperatively, the X-ray examination of the Empty group revealed the presence of bone scabs at the broken ends, though significant bone defects persisted until 12 W (Figure 4(Aa1–Aa4)). In the P group, a rough and blurred high-brightness shadow was observed around the brace at 4 W. In comparison, a lamellar high-density shadow was observed at the critical-size bone defect at 12 W, albeit with a lower density than the surrounding normal bone. Additionally, a blurred fracture line observed at the fracture end indicated the formation of new bone scabs (Figure 4(Ab1–Ab4)). The PI group showed similar image performance as the P group in the 12 W (Figure 4(Ac1–Ac4)). The PC group demonstrated complete filling of the defect with low-light images at 8 W, confirming the presence of a new bone crust. At 12 W, an increase in the density of the bone crust in the defect was observed, which was homogeneous and closely resembled the surrounding normal bone tissue (Figure 4(Ad1–Ad4)). In the PS group, bone scab formation was observed 4 W postoperatively, which filled the defect with a lower density than the surrounding bone. By 8 W, the bone crust had filled the fractured end, and its density had approached that of normal bone tissue with uniform density. After a further 12 W, the process of bone crust remodeling was essentially finalized, and the medullary cavity was reopened (Figure 4(Ae1–Ae4)). In the PCS group, high-bright cortical bone was formed around the fracture and connected to the fracture end at 4 W postoperatively, resulting in a high-bright shadow with uniform density in the defect. At 8 W postoperatively, the defect was reopened to the medullary cavity, and bone remodeling was essentially completed. By 12 W, the bone density had reached a level comparable to that of the surrounding bone tissue (Figure 4(Af1–Af4)).

#### 3.3.2. Micro-CT and Postoperative Mechanical Testing

At 12 W postoperatively, micro-CT analysis displayed a lack of noteworthy bone formation in the Empty group (Figure 4(Ba1,Ba2)) and no substantial decrease in defect length from the initial model preparation. Conversely, the P group showed some new bone growth into the scaffold and the formation of a lamellar cortical bone cover around it (Figure 4(Bb1,Bb2)). In addition, new bone formation and growth into the scaffold was observed in the PI group, particularly at the scaffold surface (Figure 4(Bc1,Bc2)). The results indicate a significant improvement in the bone repair effect in both the PC and PS groups, as evidenced by the formation of new bone in the majority of scaffolds and the successful establishment of robust bony connections at the fracture ends (Figure 4(Bd1,Bd2), Figure 4(Be1,Be2)). Notably, the PCS group displayed the most impressive repair effect, with new bone growth observed both internally and externally of the scaffold, as well as the establishment of bony connections at the fracture ends and the formation of a cortical bone layer surrounding the scaffold (Figure 4(Bf1,Bf2)).

Quantitative analysis of the area of interest revealed that the PCS group exhibited the highest BV value. Additionally, the material groups were found to be statistically different from the Empty group, with results in accordance with the imaging observations (Figure 4(Ca)). Notably, the BV/TV values indicated significant new bone formation in the PCS group (Figure 4(Cb)). Upon comparison with the remaining five groups, it was evident that the PCS group exhibited the highest count of newly formed bone, resulting in the greatest number of bone trabeculae, the most substantial thickness, and the shortest trabecular spacing. This further substantiates the superiority of the PCS group in facilitating the repair of critical-size bone defects (Figure 4(Cc–Ce)). There were no statistically significant differences observed among all groups with respect to BMD pertaining to the formation of new bone (Figure 4(Cf)).

**Figure 5:**
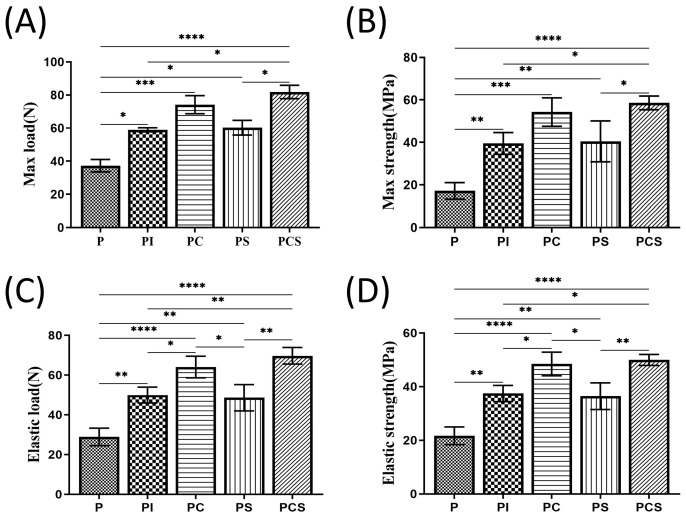
In vivo biological testing: Results of postoperative biomechanical tests performed on different functionalized groups of materials. (**A**) Max load (N); (**B**) Max strength (MPa); (**C**) Elastic load (N); (**D**) Elastic strength (MPa). The results were expressed as mean ± standard error of the mean (*n* = 3; significance levels were established as * *p <* 0.05, ** *p <* 0.01, *** *p <* 0.001, **** *p <* 0.0001).

Finally, a biomechanical examination using three-point bending was conducted 12 W after the operation. The resulting Max load, Max strength, Elastic load, and Elastic strength were computed (Figure 5A–D). The Max load results indicated that the PCS group had the highest values among all functionalized scaffolds, followed by the PC group, PS group, PI group, and P group. The Max strength, Elastic load, and Elastic strength results were similar to those of the Max load.

#### 3.3.3. Histological Testing

**Figure 6:**
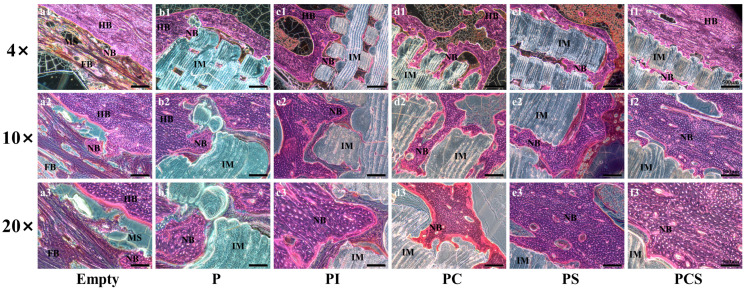
In vivo biological testing: HE Staining of 12-week rabbit critical-size bone defects in the blank control and five different functionalized modification groups. The magnifications are 4 times, 10 times, and 20 times (scale bar: 500 μm). HB: Host Bone; NB: New Bone; FB: Fibrous Tissue; IM: Implant Material; MS: Medullary Space.

The results of HE staining revealed that the bone defect region in the Empty group was occupied by fibro-soft tissue, with some instances of punctate new bone growth into the medullary cavity. In the P group, new bone formation was observed at the interface between the scaffold and the bone, with new bone growth occurring within the scaffold gap. Comparatively, the PI group exhibited a greater degree of new bone formation, with a thicker layer of new bone tissue forming around the scaffold. Additionally, partial degradation of the scaffold at the contact end was observed. The PC and PS groups demonstrated a higher degree of bone formation compared to the PI group. Furthermore, the deeper portion of the scaffold displayed the presence of new bone tissue, which grew around the scaffold and assumed an irregular shape following scaffold degradation at the contact end. Notably, the PCS group exhibited the highest level of new bone formation, with the majority of the new bone tissue being woven bone. Additionally, some of the woven bone underwent complete remodeling and formed a cortical bone-like structure by connecting with the cortical bone at the broken end. Furthermore, the scaffold’s interior was infused with nascent osseous tissue that gradually extended along the scaffold and integrated into fragments, while a portion of the scaffold underwent degradation in the region of new bone generation. However, the majority of the scaffold’s architecture persisted (Figure 6).

**Figure 7:**
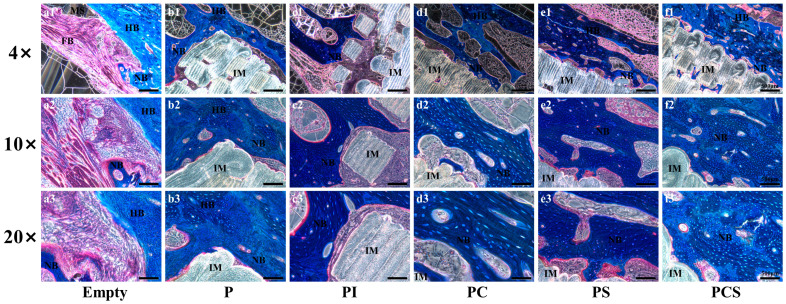
In vivo biological testing: Toluidine Blue staining of 12-week rabbit critical-size bone defects in the blank control and five different functionalized modification groups. The magnifications are 4 times, 10 times, and 20 times (scale bar: 500 μm). HB: Host Bone; NB: New Bone; FB: Fibrous Tissue; IM: Implant Material; MS: Medullary Space.

**Figure 8:**
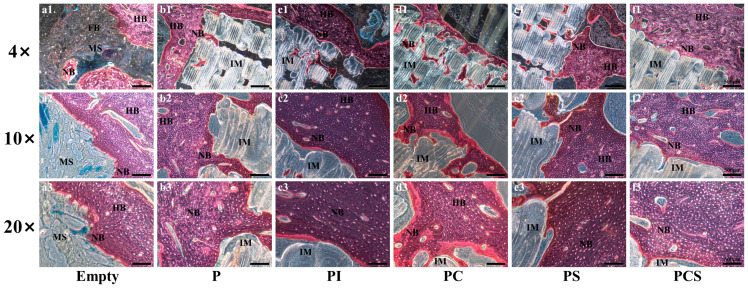
In vivo biological testing: Masson staining of 12-week rabbit critical-size bone defects in the blank control and five different functionalized modification groups. The magnifications are 4 times, 10 times, and 20 times (scale bar: 500 μm). HB: Host Bone; NB: New Bone; FB: Fibrous Tissue; IM: Implant Material; MS: Medullary Space.

The results of Toluidine blue staining and Masson staining were consistent with the results of HE staining, indicating the greatest extent of degradation and the best osseointegration effect at the contact end of the scaffold and bone in the PCS group. Moreover, the bone tissue formed outside the scaffold was arranged in an orderly manner to form a lamellar, lamellar-like cortical bone structure, and the color results showed varying shades, indicating that the new bone tissue was transforming into mature bone tissue (Figure 7 and Figure 8).

## 4. Discussion

In this study, we successfully fabricated PLA scaffolds with a porosity exceeding 80% and a pore size of 350 μm using 3D printing technology. It is well known that autologous bone grafting is widely accepted as the preferred method for treating critical-size bone defects, typically consisting of cancellous bone characterized by a porous structure and osteoinductive properties, featuring pore sizes ranging from 100 to 500 μm [8,25]. Additional research has revealed that scaffolds with a porosity exceeding 80% and pore sizes between 250 and 500 μm can promote better vascularization, nerve infiltration, and cell migration [26,27]. Therefore, this 3D-printed scaffold exhibits favorable pore size and porosity, thereby establishing a foundation for subsequent exploration of functionalized modification approaches.

The optimal scaffold for bionic bone tissue engineering must take into consideration both the structural and compositional aspects of bone in order to achieve both structural and compositional bionic [28,29]. This strategy has been shown to enhance osteoblast growth (osteoconduction) [30], ensure stable anchoring of the scaffold to host bone tissue (osseointegration) [31], and stimulate the differentiation of immature host cells into osteoblasts (osteoinduction) [32,33], ultimately promoting bone formation. Poly(lactic acid) (PLA), a medical polymer material approved by the FDA, exhibits excellent biosafety and thermoplasticity, facilitating rapid molding and curing for 3D printing. However, due to its hydrophobicity and a lack of biologically recognizable sites on its surface, alterations are necessary to improve its biocompatibility and osteogenic characteristics [34,35]. Xue et al. managed to accelerate the deposition of hydroxyapatite and calcium salts by augmenting the negative charge and chemical groups on the surface of PLGA materials through plasma treatment. This approach was found to enhance cell adhesion and proliferation, as confirmed by co-culturing with OCT-1 osteoblast-like cells [36]. Research conducted by Mohammad and colleagues has demonstrated that plasma surface modification of polyhydroxybutyrate results in increased hydrophilicity and cytocompatibility of surface nanofibers in comparison to unmodified polyhydroxybutyrate [37]. Plasma surface modification technology can alter the surface properties and microstructure of scaffolds, including roughness, morphology, charge, chemical composition, surface energy, and wettability. These alterations are achieved through processes such as etching, chemical reaction, and ion radiation. This modification can be achieved without compromising the intrinsic properties of 3D-printed polymer scaffolds, thereby facilitating effective polymer-tissue interaction [36,37]. This assertion is supported by our material characterization experiments and in vitro cellular experiments. The surface of the PI group demonstrated a significant increase in the -C-O- and C=O/-COOH bonds by 14.7% and 41.3%, respectively, compared to the P group. This demonstrates that appropriate oxygen plasma treatment could incorporate -C-O- and C=O/-COOH groups onto the PLA surface and increase its negative charges. It also produces peaks and valleys on its surface through the etching effect and changes the surface topography. It will be beneficial to develop a new surface modification route for enhancing the formation rate of collagen and apatite layers and shortening the incubation period. This finding aligns with the outcomes of prior research [36].

Type I collagen is a key component of the extracellular matrix in bone tissue, facilitating favorable conditions for cellular adhesion, proliferation, and differentiation. The integration of collagen into biomaterials as cell recognition molecules through coating or grafting has been extensively studied in order to enhance cellular surface affinity [38,39,40]. Hydroxyapatite, an essential inorganic constituent of bone, provides robust mechanical reinforcement to bone and exhibits favorable osteoconductivity and osteoinductive properties as a bone graft material [41]. Given that bone is a composite tissue composed of both inorganic hydroxyapatite minerals (Ca_10_(PO_4_)_6_(OH)_2_) and various organic components such as type I collagen, lipids, and non-collagenous proteins, the plasma surface modification technique has been used to create active sites and free radical groups on the scaffold. This enables the subsequent loading of collagen and coating with hydroxyapatite [42]. Material characterization studies have revealed that the functionalized modifications mentioned above do not significantly affect the overall properties of PLA materials. Additionally, the presence of characteristic elements such as Ca, P, Mg, and N in the modified materials suggests that the functionalized modifications have successfully achieved a bionic strategy for skeletal composition. Consequently, we achieved the successful fabrication of scaffold materials possessing a bionic bone structure and composition.

Furthermore, in vitro cell experiments have confirmed that the functionalized modification has augmented the biocompatibility of the material. Additionally, SEM and laser confocal microscopy have demonstrated that the cells have exhibited robust growth on the surface of the material following the aforementioned modification. The cells displayed normal and uniform morphology, with pseudopods extending and interconnecting and multiple cells aggregating into clusters. To gain a more comprehensive understanding of the osteogenic characteristics of 3D-printed PLA scaffolds, we conducted an examination and analysis of various osteogenic genes. Following plasma treatment, the structural and physicochemical properties of the PLA scaffold material were altered to effectively stimulate the expression of the aforementioned genes in MC3T3-E1 cells. Additionally, the application of type I collagen loading and hydroxyapatite coating was found to enhance the expression of these genes. Lei et al. demonstrated that the incorporation of nano-hydroxyapatite and type I collagen in the fabrication process of modified calcium phosphate repair materials resulted in the creation of a bionic surface that effectively facilitated cellular adhesion and proliferation as well as enhanced the expression of osteogenic genes in MC3T3-E1 cells [43]. Furthermore, bone mineralization serves as a key indicator of osteoblast maturation and an essential step in the formation of healthy bone tissue. The use of Alizarin red staining and quantitative analysis allows for the evaluation of the mineralization effects of MC3T3-E1 cells, thus demonstrating the pro-osteogenic differentiation potential of PLA scaffolds. After functionalized modification, the 3D-printed PLA scaffolds show favorable mineralization effects, with the PCS group exhibiting the most significant promotion of osteoblast mineralization. It is noteworthy that the formation of hydroxyapatite coatings is often weak, necessitating immersion in SBF for more than two weeks to achieve hydroxyapatite-like coatings [44,45]. In contrast, our plasma-modified mineralized coating achieves a similar outcome within a mere 24 h timeframe, thereby significantly enhancing the efficiency of composite preparation.

Ultimately, a critical-size rabbit radial bone defect model was established through animal experimentation to verify the efficacy of the material in repairing critical-size bone defects in vivo [2]. Indeed, in contrast to bone defects of smaller dimensions, critical-size bone defects necessitate alternative approaches for bone regeneration, including autologous bone grafting, allogeneic bone grafting, and bone tissue engineering. This is attributed to their surpassing the inherent healing capacity of the body, as substantiated by the outcomes of our in vivo experiments conducted on the Empty group. After four weeks of surgery, the PCS group displayed the formation of a bone crust-like structure around the scaffold. Micro-CT and histological examination revealed substantial osteogenesis, particularly the development of a layer of bone tissue on the material’s surface. These findings validate the PCS group scaffold’s effectiveness in promoting osteogenesis and the complete repair of critical bone defects in the radius. It is believed that the PCS group exhibited favorable reparative effects in the repair of critical-size bone defects through bionomics, as evidenced by improvements in both bone structure and composition. This may be attributed to the fact that bionomic normal bone tissue is comprised of a collagen hydroxyapatite structure, which promotes cell adhesion, proliferation, and differentiation [46]. Additionally, the scaffold undergoes resorptive remodeling based on its original components, which further accelerates bone repair [47].

The limitation of this study is the incomplete degradation of all materials within the 12-week timeframe. Therefore, additional modification studies of the PLA scaffold are necessary to expedite its degradation rate and align it with the bone repair duration. This will facilitate optimal repair outcomes and offer a viable clinical approach for treating critical-size bone defects.

## 5. Conclusions

The present investigation used 3D printing technology to fabricate PLA scaffolds, which were subsequently subjected to plasma surface modification. The successful preparation of bionic microsurface structures was achieved through the loading of type I collagen and the application of hydroxyapatite mineralization coating. The biocompatibility of the scaffolds was confirmed through in vitro experiments utilizing the modification strategy. The modified scaffolds demonstrated effective promotion of cell adhesion, proliferation, and differentiation, as well as the expression of ALP, OPN, Col-1, OCN, and RUNX2 in osteoblasts. The efficacy of the functionally modified scaffold in promoting bone repair for critical-size bone defects in the rabbit radius was further validated through in vivo experiments, thereby presenting a novel therapeutic strategy for addressing critical-size bone defects.

## 6. Patent

This functionalized modification technology has been authorized by the State Intellectual Property Office of China (patent number: ZL202210740535.7).

## Data Availability

Data are contained within the article.
